# A Novel Therapeutic Approach With Sodium Pyruvate on Vital Signs, Acid–Base, and Metabolic Disturbances in Rats With a Combined Blast and Hemorrhagic Shock

**DOI:** 10.3389/fneur.2022.938076

**Published:** 2022-08-05

**Authors:** Biswajit Saha, Geetaram Sahu, Pushpa Sharma

**Affiliations:** Department of Anesthesiology, Uniformed Services University of the Health Sciences, Bethesda, MD, United States

**Keywords:** blast injury, hemorrhagic shock, sodium pyruvate resuscitation, shock index, metabolic acid-base disorder, pyruvate dehydrogenase complex (PDH) activity

## Abstract

**Background:**

Blast injuries from improvised explosive devices (IEDs) are known to cause blast traumatic brain injuries (bTBIs), hemorrhagic shock (HS), organ damage, mitochondrial dysfunction, and subsequent free radical production. A pre-citric acid cycle reagent, pyruvate, is suggested to improve mitochondrial ATP production through the activation of the mitochondrial gatekeeper enzyme “pyruvate dehydrogenase complex (PDH).” Our study aimed to investigate the role of physiologic, metabolic, and mitochondrial effects of hypertonic sodium pyruvate resuscitation in rats with a combined blast and HS injury.

**Methods:**

A pre-clinical rat model of combined injury with repetitive 20 PSI blast exposure accompanied with HS and fluid resuscitation (sodium pyruvate as metabolic adjuvant or hypertonic saline as control), followed by transfusion of shed blood was used in this study. Control sham animals (instrumental and time-matched) received anesthesia and cannulation, but neither received any injury nor treatment. The mean arterial pressure and heart rate were recorded throughout the experiment by a computerized program. Blood collected at T0 (baseline), T60 (after HS), and T180 (end) was analyzed for blood chemistry and mitochondrial PDH enzyme activity.

**Results:**

Sodium pyruvate resuscitation significantly improved the mean arterial pressure (MAP), heart rate (HR), pulse pressure (PP), hemodynamic stability (Shock index), and autonomic response (Kerdo index) after the HS and/or blast injury. Compared with the baseline values, plasma lactate and lactate/pyruvate ratios were significantly increased. In contrast, base excess BE/(${\text{HCO}}_{3}^{-}$) was low and the pH was also acidotic <7.3, indicating the sign of metabolic acidosis after blast and HS in all animal groups. Sodium pyruvate infusion significantly corrected these parameters at the end of the experiment. The PDH activity also improved after the sodium pyruvate infusion.

**Conclusion:**

In our rat model of a combined blast and HS injury, hypertonic sodium pyruvate resuscitation was significantly effective in hemodynamic stabilization by correcting the acid–base status and mitochondrial mechanisms *via* its pyruvate dehydrogenase enzyme.

## Introduction

During modern wars and terrorist attacks, improvised explosive devices (IEDs) and roadside bombs are the leading causes of blast traumatic brain injuries (bTBI), death, and disabilities. Blast injury is typically encountered in the context of polytrauma, where multiple organs and body regions are injured from the sharp nails, frequently concomitant severe hemorrhage resulting in hemorrhagic shock (HS). The outcome of blast injury is therefore most often complicated. However, the patients with blast injuries are of particular concern, as there is evidence that the brains of blast-exposed casualties showed blood–brain barrier (BBB) breakdown, oxidative stress, and microglial activation (1), as well as subdural hemorrhage, cerebral contusion, and immunohistopathological changes in the brain (2). Most deaths in such scenarios occur in the pre-hospital (field) setting.

The hemorrhagic shock alone accounts for the preponderance (~60%) of deaths in patients with potentially salvageable injuries (~50%) (3). It may cause deleterious hemodynamic consequences and compromise the blood flow to some vital organs. Therefore, the combination of blast traumatic brain injury and HS is particularly onerous. Even brief episodes of hypotension and hypoxemia, clinical hallmarks of HS, double the mortality in patients with TBI. This is thought to be due to loss of cerebral autoregulation resulting in secondary ischemic insult to the already vulnerable brain. TBI, in turn, impairs neurologically mediated physiologic compensatory mechanisms that can be protective against mortality from HS.

The clinical consequences of hemodynamics and metabolic stress become evident shortly after blast injury and HS, while the complete response of secondary cellular injury takes longer due to the depleted cellular energy (ATP) stores and increased oxidative stress from the mitochondrial damage. Among the key mitochondrial metabolic enzymes, pyruvate dehydrogenase (PDH) is very sensitive to oxidative stress (4). This enzyme is especially important to the brain which relies mainly on carbohydrate metabolism. In the injured brain cells, PDH is believed to be inactive, leading to reduced glucose metabolism (5). Also, the mitochondrial dysfunction due to hypoxia after the injuries forces the cells into anaerobic metabolism, leading to the production of acid metabolites causing acidosis or acid–base disturbance. There remains a paucity of information on the nature of the acid–base disturbance and PDH activity in combined insults other than the blast injury or HS alone. There is also limited knowledge of the hemodynamic responses to a combined hemorrhage and blast injury, and their impact on the response to resuscitation in the prehospital setting. In light of this important knowledge gap regarding the distinct challenges associated with the treatment of a combined blast injury and HS in a prehospital setting, our designed study of combined injuries, therefore, cast light to answer the following questions: (1) What is the initial acid–base profile and physiological dysfunction from blast trauma and blood loss? (2) Can the degree of acid–base and physiological derangement be used to predict the mortality from the magnitude of blast injury and blood loss, or the time elapsed from injury to treatment? (3) How severe are the adverse hemodynamic and metabolic effects are from the acidosis? (4) What is the effect of combined injuries on the PDH activity used as an indicator of global oxidative stress?

From the discussion above, it is clear that optimal bTBI management often requires optimal HS management, especially during the pre-hospital phase. Recent studies have suggested that pyruvate, a natural product of the reaction in the last step of the glycolytic pathway, can improve the outcome in animal models with TBI or HS through mitochondrial mechanisms (6–9). In a pyruvate dose response study targeting the vital signs following hemorrhagic shock, our lab previously showed that sodium pyruvate 2.0 M was most effective in multiorgan failure and survival rate in HS (10). However, there is still a knowledge gap since the effects of this agent on a combined blast injury and HS have not been examined. It is, however, unclear if the combined insults are less or more amenable to novel therapies than TBI/HS alone. To address this important knowledge gap, this project, therefore, contributed to the goal of advancing the pre-hospital treatment of polytrauma casualties with blast injury and concomitant HS with the resuscitation of sodium pyruvate, and to check whether this antioxidant and an anti-inflammatory agent will improve the hemodynamics and metabolic derangements. Thus, the combined blast injury plus HS (blast injury + HS) is potentially an important new arena of therapy for use in civilian and blast polytrauma.

## Materials and Methods

All procedures were performed in accordance with the guidelines of the National Institutes of Health and were approved by the Institutional Animal Care and Use Committee (IACUC) of the Uniformed Services University of the Health Sciences (USUHS) at Bethesda, MD, USA.

### Animal Preparation and Surgical Procedures

The procedures were performed according to our established protocol. Adult, male Sprague-Dawley rats (300–350 g) were obtained from the Charles River Laboratories (Wilmington, MA, USA). The animals were anesthetized with 5% isoflurane in 100% oxygen in an anesthesia induction chamber, and the anesthesia was maintained *via* a nose cone (1.5–2% isoflurane + 100% oxygen). The depth of anesthesia was evaluated before surgery by the response to toe pinch. The spontaneously ventilating animals were instrumented by cannulating both femoral arteries and the right or left femoral vein with polyethylene tubing (PE50), secured with 4-0 silk ties. The cannulas were clamped and connected to closed stopcocks. The tail artery was cannulated to collect the baseline blood sample before any injury.

### Blast Injury

We used a well-established rat blast injury model developed in our laboratory. The animals underwent three repeated blast injuries of 20PSI at 15 min interval. Exposure to blast overpressure (BOP) generated in a laboratory shock tube with the Mylar membranes rupturing at predetermined pressure thresholds will produce structural and functional changes in the brain.

### Hemorrhagic Shock-Resuscitation Procedure

The instrumented, anesthetized, spontaneously ventilating animals underwent controlled arterial hemorrhage followed by volume/pressure limited resuscitation with hypertonic sodium pyruvate (2M) or hypertonic saline (7.5%) and then full resuscitation using the shed blood.

The right femoral artery was connected to a pressure transducer to monitor the mean arterial pressure (MAP). MAP and heart rate were monitored continuously and recorded every 5 min by computerized programming. The left femoral artery was connected with a catheter to a heparinized syringe to bleed the rat into hemorrhagic shock (HS). The left femoral vein was connected to an infusion pump for fluid/blood infusion. Core temperature was maintained at 37–37.5°C (measured with a rectal temperature probe) throughout the experiment using an electric heat pad.

### Induction of Hemorrhagic Shock

**Figure 1:**
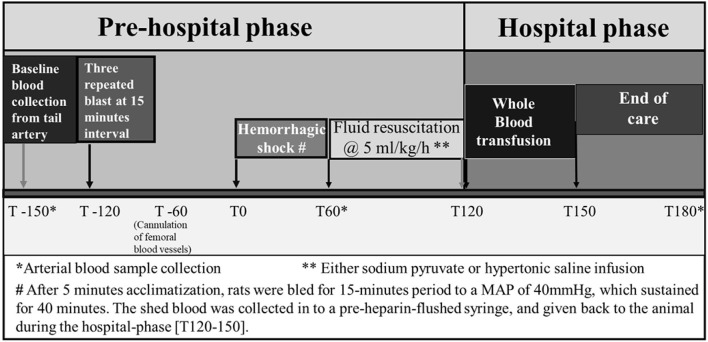
Experimental design established to conduct the study protocol of hemorrhagic shock and/or blast injury.

Rats were allowed to acclimatize for 5 min and marked as experimental time 0 (Figure 1). After circulatory variables (MAP and pulse rate) and biochemical parameters were determined, controlled hemorrhage was induced. Rats were then bled for a 15-min period to a MAP of 40 mmHg. Blood was collected in pre-heparinized tubes. MAP was sustained at 40 mmHg for 40 min by withdrawal or infusion of shed blood.

Blood collected at T0 (baseline), T60 (after HS), and T180 (end) was analyzed for blood chemistry for acid–base balance and metabolites.

Mitochondria is a major subcellular target of ischemia/ reperfusion injury, and blast injury/HS has been known to have altered mitochondrial function in various tissues. Oxidative stress has been linked to mitochondrial dysfunction and is thought to play a role in metabolic defects. As part of the mitochondrial metabolic test battery, biochemical analysis was also performed in our study to measure the plasma lactate and pyruvate levels and calculate the plasma lactate/pyruvate concentration ratio at different time-points in response to different injury and treatment groups.

### Pyruvate Assay

A total of 100 μl of fresh whole blood was added to a chilled pyruvate collection tube containing 200 μl of 8% (W/V) perchloric acid to deproteinize the blood sample. The sample was mixed well for 30 s, then placed in ice for 10 min. Centrifugation was done for 10 min at 1,500 g. Plasma pyruvate concentration was determined using a kit from Sigma, Catalog no. MAK071.

Our study also rendered the blood mitochondrial enzymatic assay for Pyruvate dehydrogenase complex (PDH) that might suggest global reduction–oxidation (redox) status, aid in prognostication, and potentially guide in using mitochondrial-targeted therapies.

### Blood-Based Dipstick Test for the Measurement of Mitochondrial Enzyme, PDH as Biomarker of Mitochondrial Damage

Blood PDH for the samples of different time-points were immunocaptured using a dipstick assay kit according to the manufacturer's guidelines (Abcam, MA, USA) using an immunologic sandwich assay. The intensity of the color band on the dipstick was measured using a flatbed scanner, and the results were analyzed by using the ImageJ software.

### Experimental Groups

Group 1. Sham (eight animals): Instrumental and time-matched sham animals received only anesthesia and cannulation but did not undergo any injury or resuscitation except the withdrawal of blood for laboratory investigation.

Groups 2 and 3. Control (eight animals in each group): Control rats received HS+/− blast injury and resuscitation with hypertonic saline (HTS) at a rate of 5 ml/kg/h. The HTS was selected as the control infusion based on the volume and osmolality matched to the hypertonic sodium pyruvate.

Groups 4 and 5. Treatment (eight animals in each group): The treatment groups underwent the injury of HS+/− blast and resuscitation with sodium pyruvate at the same rate of 5 ml/kg/h.

## Results

In this study, our major goals were to (i) examine the effects of HS on blast injury and mitochondrial damage (mechanisms) and (ii) determine whether pharmacological protection of mitochondrial impairment with sodium pyruvate can improve the outcome of injury following HS+/− blast. However, the hemodynamic changes after HS+/− blast and infusion with either 2 M sodium pyruvate (NaPyr) or 7.5% saline (HTS) have been described below.

### Sodium Pyruvate Resuscitation Improves Mean Arterial Pressure in Response to a Combined Blast and HS Injury

**Figure 2:**
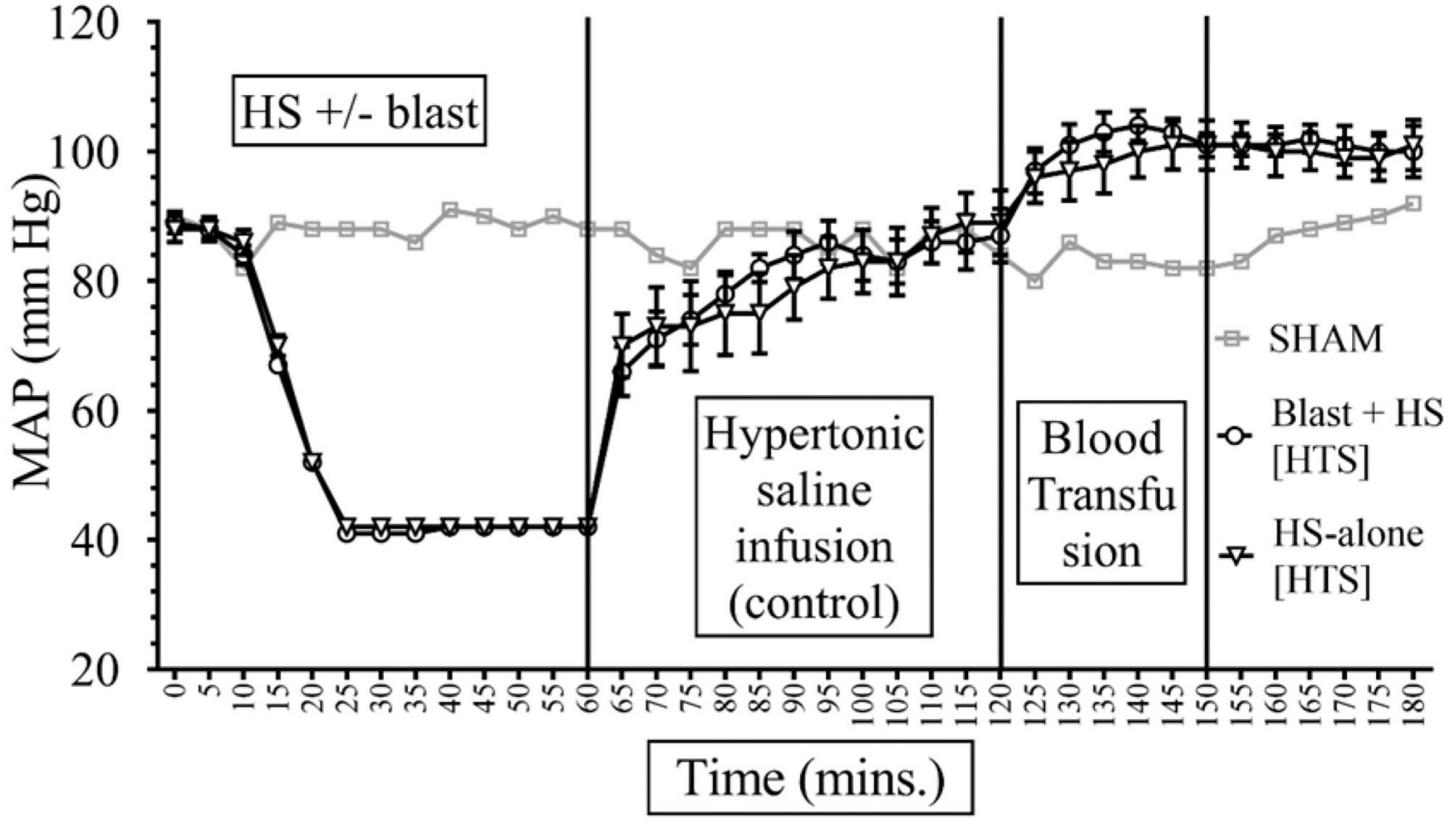
Comparison of Mean Arterial Pressure (MAP) in Mean ± SE between the injury groups of Hypertonic Saline (7.5% saline) infusion (*n* = 8). Assuming that sham represents the normal MAP values for an anesthetized rodent. All rodents had similar MAP during the baseline and HS periods of the experiments. During the HS, the MAP was maintained within 40 s as intended. Intravenous fluid (IVF) infusion of HTS increased MAP toward the normal.

**Figure 3:**
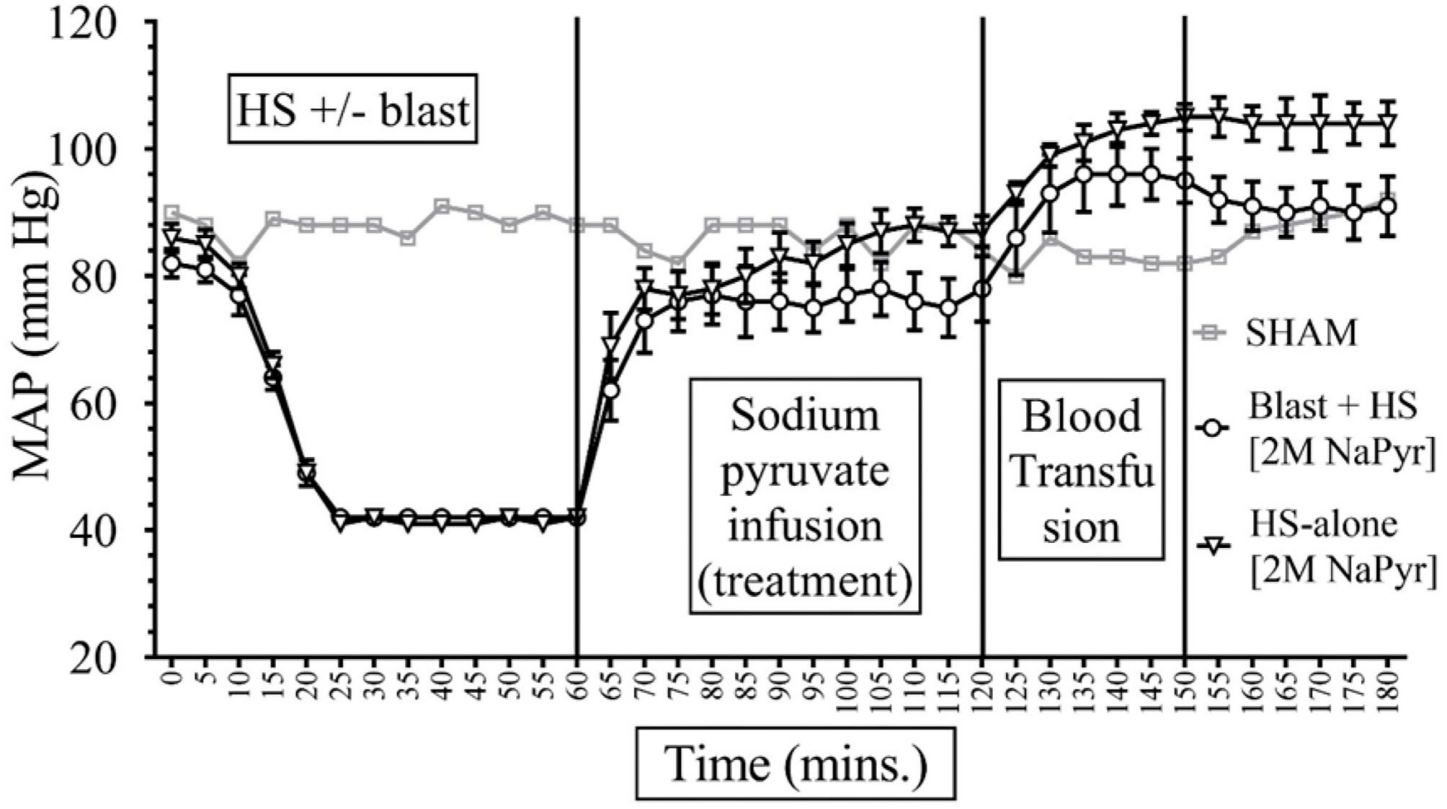
Comparison of Mean Arterial Pressure (MAP) in Mean ± SE between the injury groups of 2M NaPyr infusion (*n* = 8). NaPyr infusion increased the MAP near the baseline. The most significant increase was seen in the HS-alone group in comparison to the combined injury of high-intensity blast+HS. Blood transfusion further increased the MAP above the baseline.

Data depicted in Figures 2, 3 show that MAP was similar in all animals at the baseline (85–90 mmHg) and decreased to ~40 mmHg in all hemorrhaged groups during the 15–20-min period that put them into HS. The HS was maintained for a total duration of 60 min. Because of the similar weights of the rats, the total shed blood volume did not differ significantly among the different rats (8.42 ± 0.15 mL of blood loss). Mean arterial pressure was measured at 0–180 min in all animals, and differences were noted among the different injury and treatment groups as follows: (i) *Sham animals:* the MAP of the sham group receiving no treatments remained stable between 80 and 92 mmHg throughout the experiment. (ii) *Sodium pyruvate or hypertonic saline infusion in the injured animals (T60–120):* Both pyruvate and hypertonic saline infusions were equally effective in increasing the MAP to or near the baseline value after the HS+/− blast injury. We found a statistically significant (*p* < 0.0001) increase in MAP after HS during the pyruvate or HTS resuscitation. MAP reached a maximum of 78 ± 5 mmHg in the blast + HS group and 88 ± 3 mmHg in the HS-alone group after the pyruvate infusion. Similarly, HTS infusion increased the MAP to the maximum of 87 ± 5 mmHg in the blast + HS animals and 89 ± 5 mmHg in the HS-alone animals. It was noticeable that in comparison to the single injury group with HS alone, the combined injury groups of blast and HS had less increase of MAP during the resuscitative period with sodium pyruvate or hypertonic saline solution. (iii) *Blood transfusion that followed either the sodium pyruvate or hypertonic saline infusion (T120–180):* In our study, we found that blood transfusion was effective in stabilizing the MAP above baseline till the end in all injury and treatment groups. Following both pyruvate and blood infusions, MAP overshot the baseline to 96 ± 5 mmHg in the blast + HS group and 105 ± 3 mmHg in the HS-alone group. Similarly, MAP reached a maximum of 104 ± 3 mmHg in the blast + HS animals and 101 ± 4 mmHg in the HS-alone group after both HTS and blood infusions.

### Sodium Pyruvate Resuscitation Improves Heart Rate in Response to a Combined Blast and HS Injury

The baseline measurement (0–5 min) of HR was similar in all rats [345–375 beats per minute (bpm)]. HR decreased to approximately 325 ± 25 bpm during the 60-min period of HS. The HR of the sham animals remained stable between 350.0 ± 5 and 420 ± 5 bpm throughout the experiment. In the pyruvate-treated animals, HR increased to the maximum of 410 ± 12 bpm in the post-blast+HS group and 412 ± 10 bpm in the post-HS-alone group (*p* < 0.0001). Similarly, in the animals infused with HTS, HR reached a maximum of 378 ± 8 bpm in the post blast+HS group and 439 ± 25 bpm in the post-HS-alone group (*p* < 0.0001). However, *the blood transfusion that followed either the sodium pyruvate or hypertonic saline infusion (T120–180)* did not significantly change the HR from those associated with the intravenous fluid infusions.

### Sodium Pyruvate Resuscitation Improves Pulse Pressure in Response to a Combined Blast and HS Injury

The compensatory increase in the heart rate in response to volume loss cannot be used alone to indicate the need for hemorrhage control since it varies widely and can be changed by other variables after injury, including stress and pain. Therefore, when the pulse pressure continues to narrow, it can be used additionally as an indicative of HS, which means that the stroke volume is low. Pulse pressure was similar in all animals in our experiments at the baseline (44–48 mmHg) and decreased to ~22 ± 1 mmHg during the 60-min period of hemorrhage. Pulse pressure was measured at the T0-180-min mark and differences were noted among the different injury groups and fluid infusions. The PP of the sham group remained stable between 44 ± 1 and 50 ± 1 mmHg throughout the experiment. Sodium pyruvate solution increased the PP in the blast + HS group to the maximum of 42 ± 3 mmHg, while in the HS-alone group, it reached 47 ± 2 mmHg. After resuscitation with the HTS, PP increased to 47 ± 3 mmHg in the blast + HS animals and 48 ± 3 mmHg in the HS-alone injured animals. We found that the increase in PP after each fluid infusion was statistically significant (*p* < 0.0001) and inversely proportional to the severity of the injury, i.e., the combined injury group had less increase in comparison to the single injury group, though not statistically significant. With the blood infusion that followed either the sodium pyruvate or hypertonic saline infusion, the PP of all groups exceeded the baseline value. After both pyruvate and blood infusions, the PP increased to 57 ± 2 mmHg in the HS-alone group and 52 ± 3 mmHg in the blast + HS group. Pulse pressure increased to 55 ± 2 mmHg in the HS-alone group and 56 ± 2 mmHg in the blast+HS group after the HTS and blood infusions.

### Sodium Pyruvate Resuscitation Improves Shock Index in Response to a Combined Blast and HS Injury

**Figure 4:**
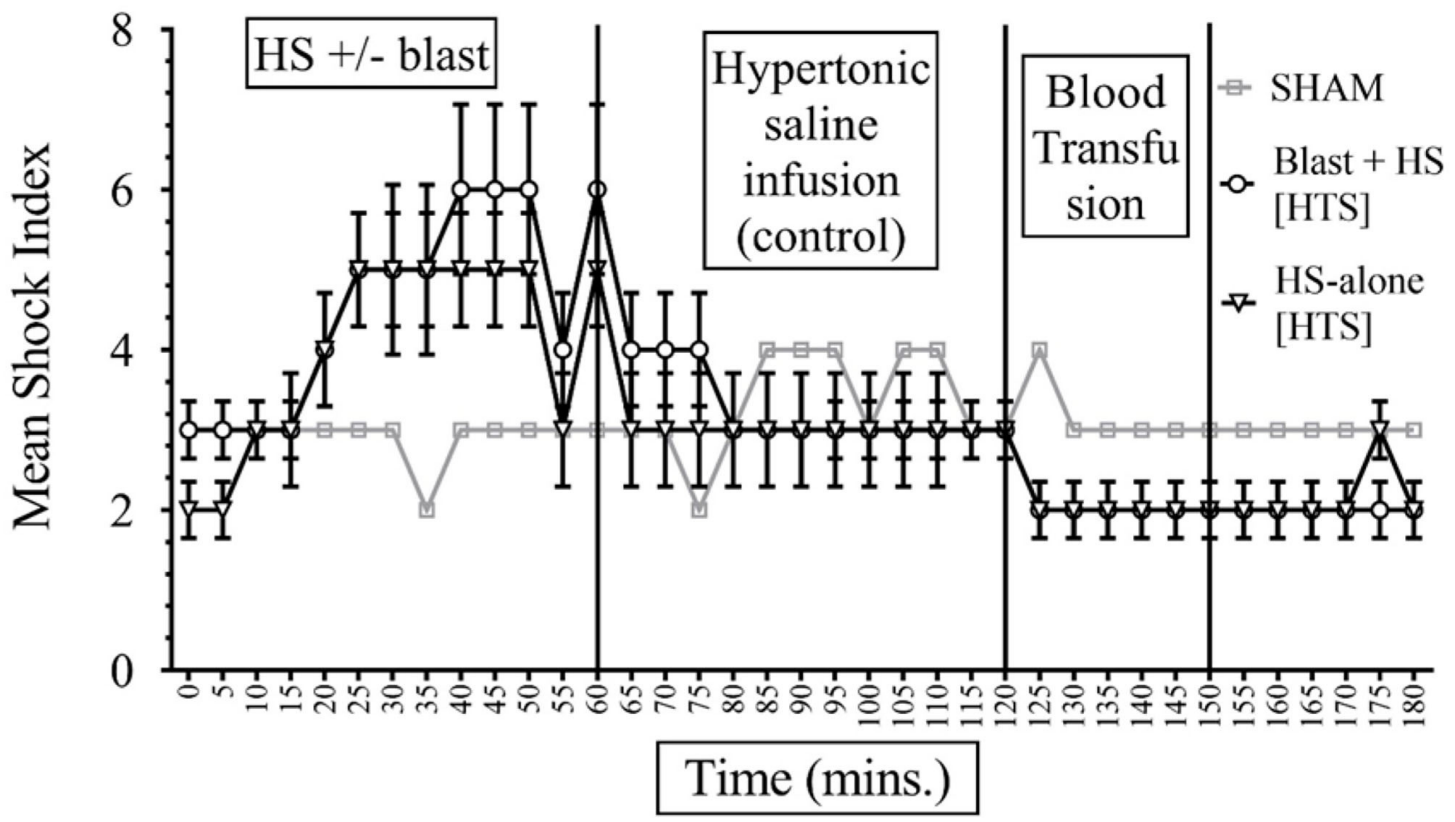
Shock Index (SI) in HS+/− blast injury animals with hypertonic saline (7.5% saline) infusion. Results are in mean ± SE, eight animals per group. SI was higher in the combined injury group in comparison to the HS-alone group after the injury. Hypertonic saline resuscitation decreased the SI to or near the baseline value.

**Figure 5:**
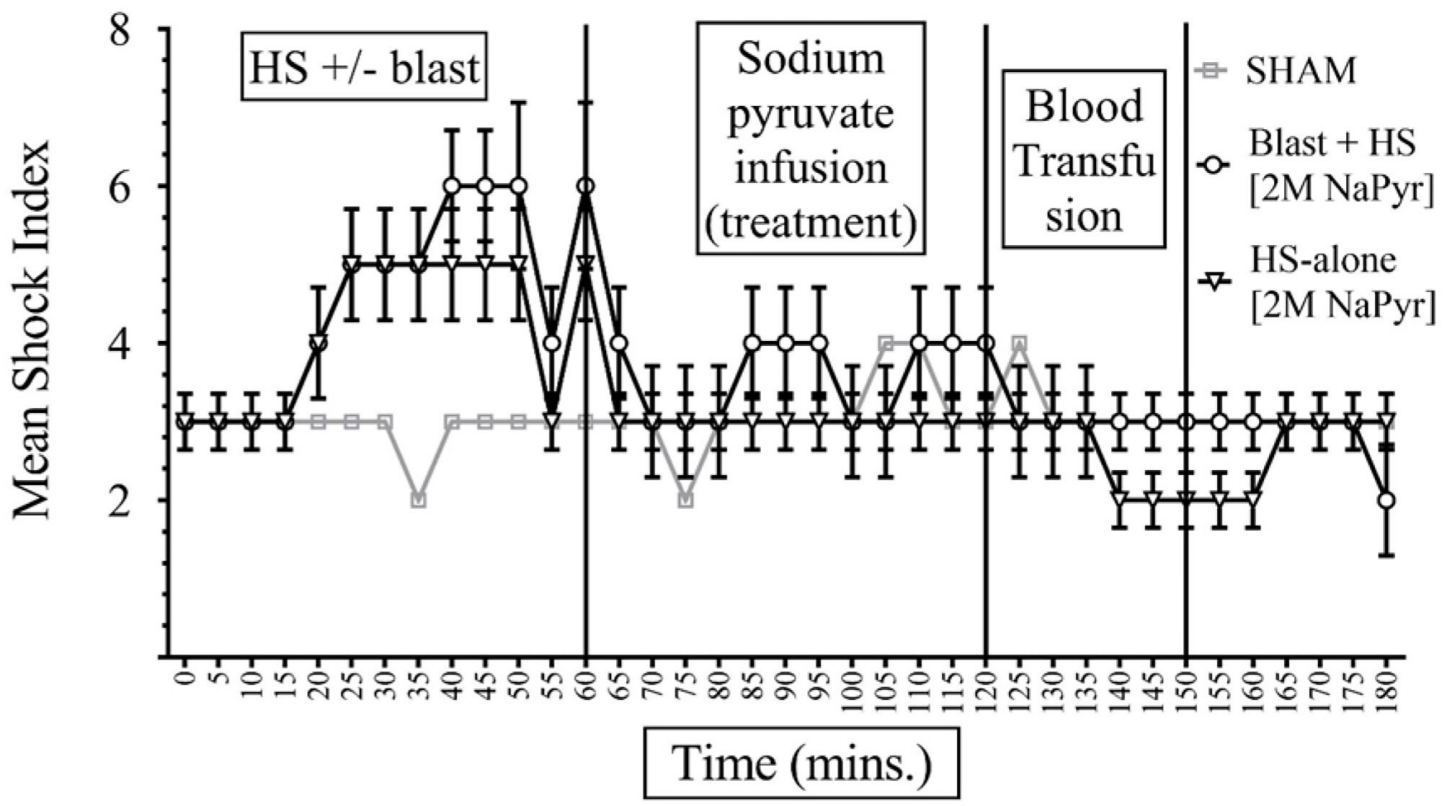
Shock Index (SI) in HS+/− blast injury animals with 2M NaPyr infusion. Results are in Mean ± SE, eight animals per group. SI was higher in the combined injury group in comparison to the HS-alone group after the injury. After the sodium pyruvate resuscitation, SI decreased better near to the baseline value in the HS-alone group in comparison to the combined injury group.

The formula of SI calculation is: [(HR in beats per minute)/(SBP in mmHg)]. In interpreting results, the normal is 0.5–0.7; elevated SI: (>0.9) was found helpful by Rady et al. to identify patients in the emergency care units requiring admission and/or intensive care despite apparently stable vital signs (11). In our study, data presented in Figures 4, 5 show that the baseline measurement (0–5 min) of SI was similar in all groups (2, 3). The highest increase of SI to 6 ± 1 was noticed in the combined injury of blast injury +HS group during the HS. We noticed that the increase in SI was directly proportional to the severity of the injury. The SI of the sham group remained stable between 2 and 3 throughout the experiment. Both sodium pyruvate and hypertonic saline infusions brought the SI to or near baseline value, which was statistically significant (*p* < 0.0001). The pyruvate infusion decreased the SI to 4 ± 0.65 in the blast injury +HS group and 3 ± 0.46 in the HS-alone group. Hypertonic saline infusion decreased the SI to 3 ± 0.6 in both blast injury +HS and HS-alone groups. The blood transfusion that followed each solution further reduced or maintained the SI at the baseline value.

### Sodium Pyruvate Resuscitation Improves the Kerdo Index in Response to a Combined Blast and HS Injury

The KI can be calculated by using the equation (1 – diastolic blood pressure/heart rate) + 100. The KI values above 100 indicate sympathicotonia and values below 100 indicate parasympathicotonia (12). In our study, KI was similar in all animals at the baseline (between 100.79 ± 0.004 and 100.81 ± 0.006) and increased to ~100.92 ± 0.005 during the HS. The KI of the sham group remained stable between 100.77 ± 0.001 and 100.80 ± 0.001 throughout the experiment. After resuscitation either with the sodium pyruvate or hypertonic saline solution, the KI decreased to the near baseline value. Sodium pyruvate solution decreased the KI the most by −0.09 in the HS-alone group and −0.08 in the blast injury +HS group. Hypertonic saline solution decreased the KI by −0.08 in both blast injury +HS and HS-alone groups. With the blood infusion that followed either the sodium pyruvate or hypertonic saline infusion, all values further decreased at or below the baseline level.

### Analysis of Blood Chemistry and Mitochondrial Enzyme

#### Sodium Pyruvate Resuscitation Improves Plasma Base Excess

Due to the volume deficit after the hemorrhagic shock, our results demonstrated a negative base excess (base deficit) https://www.sciencedirect.com/topics/medicine-and-dentistry/hypoxemia in all of the animals. We noticed an increasing severity of base deficit in the combined injury group of blast injury +HS in comparison to the HS-alone group. We found a decrease in BE/(${\text{sHCO}}_{3}^{-}$) associated with ongoing hemorrhage, which was more, i.e., a 6-fold decrease and statistically significant (*p* < 0.001) in the blast+HS group (Table 1). Along with the base deficit, the pH was also acidotic, which means that the primary process was metabolic acidosis. In addition, the PCO_2_ value decreased from the baseline value during hemorrhage, which also means that the process was metabolic acidosis with compensatory respiratory alkalosis (Table 2). This metabolic acidosis was corrected at the end of the experiment after fluid infusion/blood transfusion. But when we compared the sodium pyruvate infusion groups with the hypertonic saline infusion, we noticed sodium pyruvate solution more effectively corrected that metabolic acidosis, which was a 2- to 4-fold increase in BE/(${\text{sHCO}}_{3}^{-}$) after HS and statistically significant (*p* < 0.001) too. Sham animals did not have any noticeable changes in the acid–base status during the experiment.

**Table 1:** Acid–base balance in mean ± SE.

**Parameters**	**Groups**	**Time points**		
		**Baseline**	**End of hemorrhage**	**End of resuscitation**
P H	Sham	7.41 ± 0.02	7.43 ± 0.01	7.38 ± 0.01
	Blast injury +HS (*n* = 8) (7.5% Saline infusion)	7.18 ± 0.03	7.02 ± 0.03	7.1 ± 0.02
	Blast injury + HS (*n* = 8) (2M NaPyr infusion)	7.18 ± 0.01	7.06 ± 0.02	7.22 ± 0.04
	HS-alone (*n* = 8) (7.5% Saline infusion)	7.1 ± 0.03	7.04 ± 0.03	7 ± 0.09
	HS-alone (*n* = 8) (2M NaPyr infusion)	7.03 ± 0.02	7.04 ± 0.02	7.27 ± 0.04
BE (mmol/L)	Sham	2.67 ± 0.93	−0.89 ± 0.72	5.44 ± 1.06
	Blast injury + HS (*n* = 8) (7.5% Saline infusion)	1 ± 0.73	−9.83 ± 1.11^*^	0.33 ± 0.76
	Blast injury + HS (*n* = 8) (2M NaPyr infusion)	2.25 ± 0.7	−10.13 ± 1.2^*^	12.63 ± 3.39∧
	HS-alone (*n* = 8) (7.5% Saline infusion)	−6.17 ± 0.83	−11 ± 2.14	0 ± 0.91
	HS-alone (*n* = 8) (2M NaPyr infusion)	−7 ± 1.52	−6.88 ± 1.23	19.14 ± 0.97∧
HCO3 (mmol/L)	Sham	27.22 ± 0.83	23.32 ± 0.57	31.71 ± 0.86
	Blast injury + HS (*n* = 8) (7.5% Saline infusion)	29.52 ± 0.73	20.87 ± 1.13^*^	29.82 ± 0.89
	Blast injury + HS (*n* = 8) (2M NaPyr infusion)	30.58 ± 0.53	20.24 ± 1.12^*^	40.46 ± 2.91∧
	HS-alone (*n* = 8) (7.5% Saline infusion)	23.47 ± 0.59	19.88 ± 2.01	29.82 ± 1.03
	HS-alone (*n* = 8) (2M NaPyr infusion)	23.66 ± 1.38	23.36 ± 1.32	45.81 ± 0.83∧

*Results indicated that there was metabolic acidosis in all animals after HS, especially more in the combined injury group.*

* *p < 0.001, when compared with “baseline value” of the same group.*

∧ *p < 0.001, when compared with the “end of hemorrhage” value of the same group.*

**Table 2:** Blood gases in mean ± SE.

**Parameters**	**Groups**	**Time points**		
		**Baseline**	**End of hemorrhage**	**End of resuscitation**
PCO2 (mmHg)	Sham	43.56 ± 2.37	40.86 ± 1.62	54.88 ± 3.32
	Blast injury + HS (*n* = 8) (7.5% saline infusion)	81.05 ± 7.47	71.6 ± 8.8	95.97 ± 5.66
	Blast injury + HS (*n* = 8) (2M NaPyr infusion)	81.56 ± 2.06	72.19 ± 4.43	102.06 ± 7.69
	HS-alone (*n* = 8) (7.5% saline infusion)	75.77 ± 4.74	74.68 ± 9.35	104.08 ± 7.25
	HS-alone (*n* = 8) (2M NaPyr infusion)	89.26 ± 8.05	88.53 ± 5.49	102.93 ± 8.92
PO2 (mmHg)	Sham	360.17 ± 16.60	326.22 ± 19.81	414.89 ± 23.09
	Blast injury + HS (*n* = 8) (7.5% saline infusion)	305 ± 13.61	309.17 ± 14.25	408.83 ± 12
	Blast injury + HS (*n* = 8) (2M NaPyr infusion)	354.38 ± 14.87	298.38 ± 8.75	358.75 ± 14.89
	HS-alone (*n* = 8) (7.5% saline infusion)	308.17 ± 6.96	317 ± 15.67	410.67 ± 40.53
	HS-alone (*n* = 8) (2M NaPyr infusion)	313.75 ± 8.09	323.88 ± 19.6	397 ± 18.81
ETCO2 (mmol/L)	Sham	28.50 ± 0.87	24.44 ± 0.58	33.33 ± 0.92
	Blast injury + HS (*n* = 8) (7.5% saline infusion)	31.83 ± 0.87	23.33 ± 1.38	32.83 ± 1.05
	Blast injury + HS (*n* = 8) (2M NaPyr infusion)	33 ± 0.5	22.38 ± 1.32	43.38 ± 2.95
	HS-alone (*n* = 8) (7.5% saline infusion)	25.83 ± 0.65	22.17 ± 2.18	32.8 ± 1.17
	HS-alone (*n* = 8) (2M NaPyr infusion)	26 ± 1.49	26.38 ± 1.5	48.29 ± 0.67

*The results are (Mean ± SE) of blood gases of eight animals per group in response to blast/HS and resuscitation. The PCO_2_ value decreased from the baseline value after HS to compensate metabolic acidosis with compensatory respiratory alkalosis*.

### Sodium Pyruvate Resuscitation Improves Plasma Lactate

The changes in mitochondrial activity after the trauma and the possibilities of rapid progression through a secondary cell injury cascade necessitate different therapies to be studied to limit the mitochondrial damage and retain/restore its function after TBI/HS. As mentioned earlier, we aimed to demonstrate the potential therapeutic value of sodium pyruvate in our protocol, which will mediate mitochondrial protection in secondary brain injury. We noticed that the lactate concentration increased after the injury in all blast + HS and HS-alone groups, but when compared between the groups, this was more in the blast + HS animals. This lactate increase after HS was 5-fold and statistically significant (*p* < 0.001) from the baseline values in all blast + HS animal groups. The lactate concentration decreased at the end of experiments after both intravenous fluid infusions with no superiority to each other. So, the addition of pyruvate in this model resulted in less acidosis and lower lactate-level, indicating that the pyruvate was shunted toward aerobic respiration (Table 3).

**Table 3:** Blood metabolites in mean ± SE.

**Parameters**	**Groups**	**Time points**		
		**Baseline**	**End of hemorrhage**	**End of resuscitation**
Glucose (mg/dL)	Sham	172 ± 4.88	139.75 ± 0.28	158.00 ± 4.63
	Blast injury + HS (*n* = 8) (7.5% saline infusion)	232.33 ± 11.37	354.33 ± 22.63^*^	191.5 ± 9.87∧
	Blast injury + HS (*n* = 8) (2M NaPyr infusion)	257.38 ± 10.53	345.13 ± 25.62^*^	178.5 ± 14.33∧
	HS-alone (*n* = 8) (7.5% saline infusion)	167.17 ± 10.2	271.33 ± 32.86^*^	148.67 ± 20.76∧
	HS-alone (*n* = 8) (2M NaPyr infusion)	158.75 ± 8.94	294.88 ± 20.14^*^	175.71 ± 7.94∧
Lactate (mmol/L)	Sham	1.01 ± 0.20	0.76 ± 0.04	1.17 ± 0.08
	Blast injury + HS (*n* = 8) (7.5% saline infusion)	0.62 ± 0.08	2.26 ± 0.18^*^	0.79 ± 0.18∧
	Blast injury + HS (*n* = 8) (2M NaPyr infusion)	0.54 ± 0.04	2.48 ± 0.55^*^	1.60 ±0.31
	HS-alone (*n* = 8) (7.5% saline infusi on)	0.59 ± 0.08	2.09 ± 0.60^*^	0.65 ± 0.18∧
	HS-alone (*n* = 8) (2M NaPyr infusion)	0.68 ± 0.11	1.66 ± 0.38	1.14 ± 0.17

*Data are presented as mean + standard error of the mean (SEM), n = 8 animals in each group. Animals with combined blast and HS had both higher lactate and glucose levels compared with single injury.*

* *p < 0.001, when compared with “baseline value” of the same group.*

∧ *p < 0.001, when compared with the “end of hemorrhage” value of the same group*.

#### Sodium Pyruvate Resuscitation Improves Lactate Clearance

In our experiments, lactate clearance was calculated using the following formula: lactate clearance = (lactate_afterHS_–lactate_endvalue_)/lactate_afterHS_ × 100 (expressed as percentage change after the injury). We found a positive lactate clearance indicating a decrease of lactate over time in the surviving blast injury + HS and HS-alone groups after each fluid infusion/treatment, with the largest decrease in lactate (>60%) over 2 h. In animals with continuously increased lactate levels, there was no lactate clearance, which caused them to die early. So, we could conclude that lactate clearance may potentially be a useful tool to predict mortality, as lower lactate clearance is associated with increased mortality.

#### Sodium Pyruvate Resuscitation Improves Plasma Lactate/Pyruvate Ratio

Analysis of pyruvate along with lactate is of diagnostic importance that may suggest any error of metabolism as some present with lactic acidosis and/or a high lactate-to-pyruvate (L/P) ratio, a measure of NADH/NAD+ redox state in the circulation. In our experiments, we did not find any noticeable changes in the pyruvate levels at any time-points in different injury and treatment groups.

**Figure 6:**
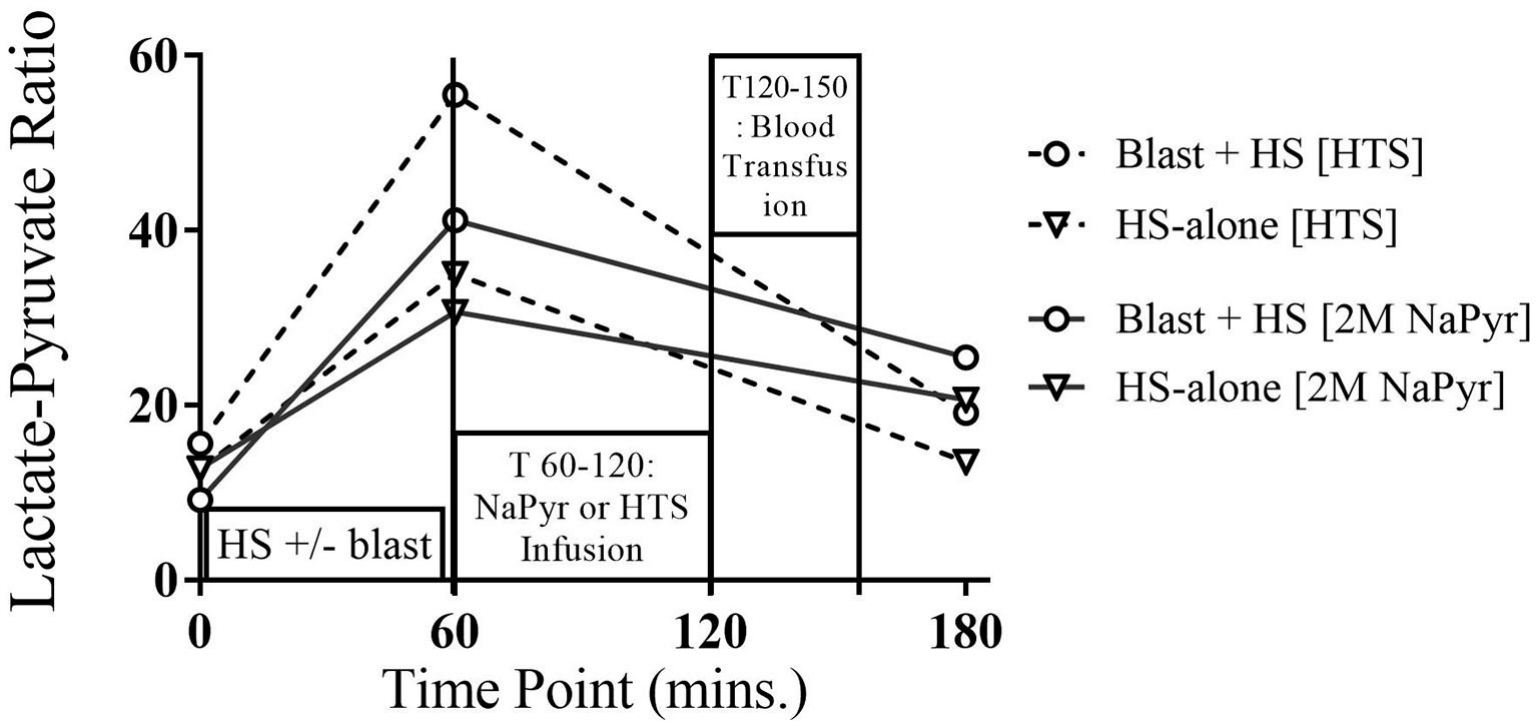
Plasma lactate:pyruvate of 4-h survival animals at different time-points of HS and/or 20 PSI blast injury, and with either sodium pyruvate (treatment) or hypertonic saline (control) infusion. There was a statistically significant (*p* < 0.001) increase in the L:P ratio after the combined blast and HS injury (T60) from the baseline value. The condition greatly improved after both infusions.

Usually, an L:P ratio >25 is considered increased and suggestive of a primary (or secondary) respiratory chain dysfunction and resultant production of the toxic reactive oxygen species (ROS). In our experiments, this condition occurred, i.e., the L/P ratio in blood was above 25 in all instances after the HS due to hypoxia, especially more and statistically significant (*p* < 0.001) changes from the baseline when this HS was combined with the highest intensity (20 PSI) blast injury (Figure 6). Here in our experiments, the increased L/P ratio was mainly due to the increased lactate levels as there were no noticeable changes in the plasma pyruvate levels. Fluid infusions of all treatment and control solutions effectively decreased the L/P ratio at the end of the experiment, indicating that the pyruvate was shunted toward aerobic respiration.

#### Sodium Pyruvate Resuscitation Improves Hyperglycemia

Our study showed an altered energy metabolism after the injury. We noticed an almost 2-fold increase in total blood glucose levels after the injuries of HS and/or blast because there was a limited amount of oxygen, and hence might promote the anaerobic glycolysis. In our experiments, after infusion of sodium pyruvate or hypertonic saline solution and blood transfusion, the increased blood glucose levels returned to the near baseline value at the end (Table 3).

#### Sodium Pyruvate Resuscitation Improves PDH Activity After the Injury

Our objective behind the use of PDH activity as a biomarker of blast injury/HS is that the dysfunctional mitochondria in the injured brain results in leakage of toxic free radicals into its matrix, cytosol, and subsequently into the circulation resulting in secondary cell injury and neuronal cell death. Therefore, the consequences of blast injury/HS on oxidative stress should reflect the PDH activity in blood.

**Figure 7:**
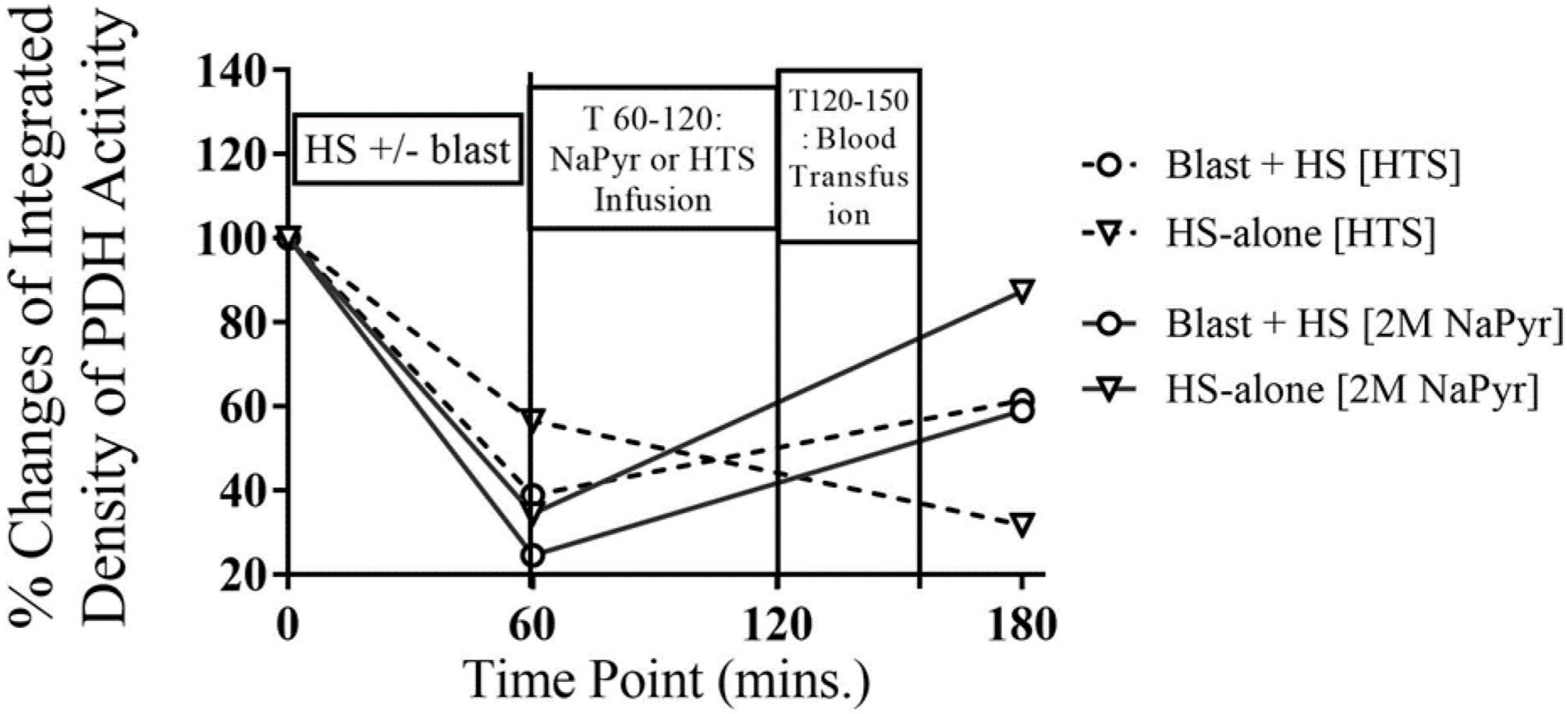
PDH enzyme activity in whole blood at different time-points of HS and/or 20PSI blast injury, and sodium pyruvate (treatment) or hypertonic saline (control) infused animals (*n* = 8) surviving for 4 h. PDH activity decreased from the baseline value in all injury groups at T60, which was not statistically significant. The PDH enzyme activity greatly improved after sodium pyruvate infusion, which was dependent on the severity of the injury. Sham animals did not show any changes that has not been mentioned here.

In our experiments, we found a 62–76% decrease in PDH activity from the baseline value after the combined injury of blast and HS. In comparison to that, there was a 43–65% decrease in PDH activity in the single injury group of HS-alone. So, the decreased activity of PDH was inversely proportional to the severity of the injury and statistically significant (*p* < 0.001) too (Figure 7). The enzyme activity improved better after the sodium pyruvate infusion in comparison to the control hypertonic saline infusion.

## Discussion

As many as 50% of civilian urban, 80% of civilian rural, and 90% of military trauma fatalities occur before hospital arrival (3, 13). Consequently, most patients do not benefit from transformational improvements in hospital trauma care that have occurred over the last decades. These data show that improving pre-hospital resuscitation outcomes should be a key priority of traumatologists, representing a major unmet medical need.

Standard pre-hospital treatment includes basic life support, hemostasis efforts, and intravenous fluid resuscitation. The main objective of fluid resuscitation is volume replacement for the lost blood, restoration of vital signs, improvement of oxygen metabolism, prevention of cell death, organ damage, and enhancement of survival. The standard high-volume isosmotic resuscitation fluids restore intravascular volume but dilute O_2_ content, further contributing to tissue hypoxia and anaerobic metabolism. Blood transfusions can be life-saving but are rarely available before hospital arrival as it requires refrigeration and typing. Consequently, mortality remains high for patients with severe HS (~1 in 2) with current standard care. Therefore, one major issue with current standards for resuscitation of HS patients with concomitant blast injury appears to be providing adequate tissue (brain and other vital organs) oxygenation/cellular respiration without exacerbating cerebral edema with hypotonic volume overload.

In this study, using a rat model of HS+/− blast, we have demonstrated that infusion of low-volume hypertonic sodium pyruvate (HSP) solution as a metabolic adjuvant is significantly better in normalizing the physiologic measures while reducing cellular injury. Administration of sodium pyruvate during the pre-hospital phase of our study hastened restitution of hemodynamic and metabolic function. Our investigation was also the first to document the effects of sodium pyruvate infusion in the setting of combined brain trauma and hemorrhagic shock.

### Intravenous Sodium Pyruvate Effects on Hemodynamics

Explosions can potentially cause massive hemorrhage, thus causing hemodynamic instability and putting a management challenge. Our study demonstrated that the hemodynamic triad of immediate bradycardia, hypotension, and increased Shock index was directly proportional to the severity of the injury, i.e., in the combined injury of blast + HS, there were more deleterious effects on hemodynamic parameters compared to the HS-alone trauma. In our hemorrhaging explosion-injured animals, after the start of the protocol infusions, both pyruvate and osmotic control groups significantly increased the MAP and HR, a measure of the myocardial contractile performance and ATP demand. These post-resuscitation improvements in hemodynamic parameters also depended on the severity of the injury. Pyruvate is a readily oxidized mitochondrial fuel that can be easily transported across the mitochondrial membrane. So, the post-resuscitation improvement in cardiac function could possibly be attributed to increased energy supply for cardiac work afforded by mitochondrial ATP production, thus resulting in enhanced myocardial energetic stability.

In this study, both the treatment and control solutions were efficacious in lowering the Shock index and the ratio of the HR to SBP. SI is a sensitive indicator of left ventricular dysfunction that can be elevated following a reduction in left ventricular stroke work (14). SI can display variability in critical patients displaying normal vital signs (15). Persistently elevated SI is also associated with poor outcomes in critically ill patients (16).

In this study, the shock induced in the combined injury of blast overpressure and hemorrhage might be multifactorial due to hemorrhagic and non-hemorrhagic causes. The severe TBI caused by repeated high-intensity (20 PSI) blast exposure might cause cardiovascular abnormalities in the animals. The hypotension might result from the disruption of brainstem centers for hemodynamic control associated with diffused axonal injury. However, hemorrhagic shock is a type of hypovolemic shock. This study developed as a result of intravascular volume loss due to bleeding out of the body, causing insufficient oxygen delivery to the cells. Restoration of intravascular volume was maintained in this study either with the sodium pyruvate or concentrated saline solution in an effort to stabilize the arterial blood pressure and protect the brain from secondary brain injury. In our study, the infusion of sodium pyruvate significantly (*P* < 0.050) increased the arterial blood pressure after the hemorrhagic shock.

The controlled resuscitation with the sodium pyruvate solution after the hemorrhagic shock introduces a potent, membrane-permeable antioxidant to the systemic circulation, which is thought to be capable of neutralizing the reactive oxygen and nitrogen species responsible for initiating the systemic inflammatory cascades (17–20). The antioxidant and anti-inflammatory capabilities of sodium pyruvate (17, 18), which is unique among the metabolic substrates, might have played a role in protecting the myocardium and possibly the vascular endothelium, thus stabilizing the systemic arterial pressure.

Acute hemorrhagic shock is known to reduce the contractility of the left ventricle. Pyruvate, a key intermediate of oxidative metabolism, has been shown to exert positive-inotropic effects on the heart and improves the contractile function as well as the hemodynamics both *in vivo* and *in vitro* (21–23). In this investigation, the improved arterial blood pressure, and, therefore, survival could probably be ascribed to (1) enhanced cardiac performance, and (2) increased cardiac tolerance to decreases in the MAP and ischemia.

Circulating pyruvate might also have helped maintain vascular performance by elevating the circulating ${\text{HCO}}_{3}^{-}$ and base excess as evidenced in these experiments, thereby affording a more favorable acid–base chemistry profile to offset acidemia resulting from end-organ hypoxia.

### Intravenous Sodium Pyruvate Effects on Blood Acid–Base Status, Lactate and Glucose Levels, and Lactate/Pyruvate Ratio

#### Metabolic Acidosis

The metabolic derangement is a key hallmark of major traumatic injury. As shown in this study, severe hemodynamic shifts were achieved due to the development of metabolic acidosis and some compensation after hemorrhagic shock. During shock, the decreased cardiac output, tachycardia, hypotension, and hypovolemic shock cause inadequate organ perfusion and oxygen delivery that interfere with aerobic metabolism. Increased anaerobic metabolism leads to the production of lactic acid and metabolic acidosis. The body's buffering capacity is reduced due to the loss of bicarbonate and hemoglobin during the hemorrhage. In our experiments, we observed statistically significant (*p* < 0.05) metabolic aberrations in plasma that were triggered by brain trauma and hemorrhagic shock, especially more in the combined injury groups in comparison to the single injury groups. This metabolic acidosis statistically significantly (*p* < 0.05) got corrected at the end of the experiment after pyruvate infusion.

*Compensatory mechanisms for acid–base disturbances and blood gases:* In the presence of metabolic acidosis after the hemorrhage, we noticed that the PCO_2_ value decreased from the baseline value, which means that the process was metabolic acidosis with compensatory respiratory alkalosis. The respiratory system responds to metabolic acidosis quickly and predictably by hyperventilation, causing more CO_2_ elimination through the stimulation of central chemoreceptors in the medulla and peripheral chemoreceptors in the carotid and aortic bodies. Over time, the respiratory compensation improved the acidosis after both fluid infusions/blood transfusion in our experiments, but to a greater extent in the pyruvate-treated animals.

#### Lactic Acidosis

For decades, lactate has been considered an excellent biomarker of oxygen limitation and organ hypoxia. This study aimed to evaluate the frequency of increased plasma lactate levels. We observed that lactic acidosis occurred at the setting of HS+/− blast, where oxygen deficiency is absolute or relative (hypoxia, ischemia, and hypotension). The combined hemorrhagic shock and blast-induced animals had a statistically significant (*p* < 0.05) higher production of lactic acid. After resuscitation with intravenous fluids, all solutions decreased the plasma lactate level near the baseline at T180.

In this study, to assess the metabolic stress after the injury, we also measured the global indexes of the cytosolic redox ratio [lactate/pyruvate (NADHc)/(NADc+)]. We noticed that the critical decreases in oxygen delivery after HS+/− blast injury caused an increase in the lactate/pyruvate ratio, and the pH dropped. These metabolic alterations were caused by both morphologic and enzymatic alterations of mitochondria in several organs. These cellular changes caused by a transient decrease in mitochondrial activity and cellular ATP could lead to secondary permanent mitochondrial damage, causing an irreversible inhibition of mitochondrial function (24). Several *in vitro* studies on liver mitochondria indicated this possibility (25). The exogenous administration of sodium pyruvate in our study sharply lowered the arterial lactate/pyruvate ratio in the face of post-injury metabolic stress. The pyruvate-induced decrease in cytoplasmic redox potential [(NADHc)/(NADc+)] bolsters the cellular phosphorylation potential [(ATP)/(ADP)(Pi)] by coupling the cytoplasmic redox state to the phosphorylation potential through the glyceraldehyde-3-phosphate dehydrogenase/phosphoglycerate kinase system (26–28). Thus, maintaining the (ATP)/(ADP)(Pi) within the physiological limits is considered essential to improve cellular function by recovering from the ischemic stress (29, 30). Mongan et al. proposed that pyruvate, by reducing metabolic acidosis and stabilizing the ATP phosphorylation potentials, may suppress the opening of KATP channels, thereby preventing a decrease in intracellular Ca^2+^, preserving the contractile function of vascular smooth muscle, and stabilizing the vasomotor tone (31–33).

#### Lactate/Pyruvate Ratio

In TBI patients, it is important to monitor the glucose delivery to the brain, and thus, using the L/P ratio as a marker of balance between the “aerobic” (referring to the TCA cycle) and “anaerobic” metabolism (referring to glycolysis culminating in lactate) (34). The best-known metabolic characteristics of an injured brain are a high lactate concentration and a high lactate/pyruvate (L/P) ratio, which are associated statistically with unfavorable clinical outcomes. When cellular respiration is impaired, as in hypoxia, pyruvate oxidation is reduced, resulting in lactic acidosis. In such situations, reduced forms of oxido-reduction coenzymes (NADH, FADH2) predominate and the L/P ratio increases. As expected, sodium pyruvate resuscitation in our experiments lowered the circulating lactate/pyruvate ratio. This can be considered to indicate the oxidation of NADH within the tissues, thus further protecting the tissue from increased oxidative stress.

#### Glucose Homeostasis

Mitochondrial dysfunction in HS+/− blast injury impairs the ability to utilize glucose effectively. Pyruvate dehydrogenase (PDH), a key enzyme to modulate glucose oxidation, significantly loses its activity during hypoxia. Sharma et al. demonstrated that TBI causes a significant reduction in PDH enzyme (5). Therefore, the altered glucose homeostasis manifested itself as initial hyperglycemia after the injury in this study. Pyruvate or hypertonic saline resuscitation improved glucose metabolism, and blood glucose levels returned to the near baseline value at the end of our experiments.

#### Pyruvate Dehydrogenase (PDH) Activity

Pyruvate dehydrogenase is a rate-limiting enzyme of the mitochondrial tri-carboxylic-acid cycle (TCA) and is an important bridge between aerobic and anaerobic respiration. The PDH is a target of oxidative stress, and its activity has been shown to undergo a significant decrease following ischemia (35). Our results demonstrated that PDH was susceptible to damage and inactivation by ROS during blast/HS.

Our study showed a statistically significant (*p* < 0.001) decrease in PDH activity after the HS+/− blast injury. Therefore, the impaired activity of PDH contributed to hyperglycemia and lactic acidosis due to reduced glucose metabolism. The sodium pyruvate treatment in our experiments after the injury improved the PDH activity. The possible mechanisms by which pyruvate was effective in preventing brain injury and loss of PDH activity may be (1) due to its antioxidant properties and (2) to optimize mitochondrial metabolism by providing substrate to PDH enzyme reaction and anaplerotic TCA cycle precursor (pyruvate carboxylase).

Here, in our experiments, the plasma pyruvate concentration did not change after the resuscitation as the improved PDH activity increased the conversion of pyruvate to acetyl-coenzyme A (acetyl-CoA), which serves as fuel for the tricarboxylic acid (TCA) cycle in the next stage of cellular respiration. In our study, we were not sure about the integrity of the electron transport chain (ETC) after the injury to be capable of generating adequate adenosine triphosphate (ATP). So, our future study direction warrants assessing the integrity of the ETC. However, our study had several limitations of not measuring the other physiological parameters [e.g., intracranial pressure (ICP)], and hence was unable to calculate the cerebral perfusion pressure (CPP). Our study also did not complement continuous brain tissue oxygen (PbtO_2_) monitoring.

In conclusion, it is clear that the dysfunction of mitochondria is the main cause of energy failure and deleterious effects and might play a pivotal role in the secondary insult after bTBI and hemorrhagic shock. The mitochondrial-targeted multipotential therapeutic agent, sodium pyruvate, may provide new hope for the treatment of blast injury and hemorrhagic states. Administration of intravenous pyruvate during the pre-hospital rescue may have a major therapeutic potential for metabolic failure, cardiovascular decompensation, and death.

## Data Availability Statement

The original contributions presented in the study are included in the article/supplementary material, further inquiries can be directed to the corresponding author/s.

## Ethics Statement

The animal study was reviewed and approved by the Institutional Animal Care and Use Committee (IACUC) of the Uniformed Services University of the Health Sciences (USUHS) at Bethesda, MD, USA.

## Author Contributions

PS contributed to the conception and design of the work, acquisition and interpretation of data, and substantial revisions. BS performed the experiments, contributed to data analysis, and significant contribution in writing the manuscript. GS performed biochemical analysis and contributed to animal experiments.

## Funding

This research was supported by a CDMRP-DM167094 grant to PS.

## Author Disclaimer

The views expressed in this article are those of the authors and do not necessarily reflect the official policy or position of the Uniformed Services University, Department of the Navy, Department of Defense, nor the U.S. Government.

## Conflict of Interest

The authors declare that the research was conducted in the absence of any commercial or financial relationships that could be construed as a potential conflict of interest.

## Publisher's Note

All claims expressed in this article are solely those of the authors and do not necessarily represent those of their affiliated organizations, or those of the publisher, the editors and the reviewers. Any product that may be evaluated in this article, or claim that may be made by its manufacturer, is not guaranteed or endorsed by the publisher.
